# Almost Being There: Video Communication with Young Children

**DOI:** 10.1371/journal.pone.0017129

**Published:** 2011-02-24

**Authors:** Joanne Catherine Tarasuik, Roslyn Galligan, Jordy Kaufman

**Affiliations:** Brain and Psychological Sciences Research Centre, Swinburne University of Technology, Melbourne, Victoria, Australia; University of Liverpool, United Kingdom

## Abstract

**Background:**

Video communication is increasingly used to connect people around the world. This includes connecting young children with their parents and other relatives during times of separation. An important issue is the extent to which video communication with children can approximate a physical presence such that familial relationships can be truly maintained by this means.

**Methodology/Principal Findings:**

The current study employed an adaptation of the Separation and Reunion Paradigm with children (17 months to 5 years) to investigate the potential for video communication with a parent to afford a sense of proximity and security to children. The protocol involved a free-play session with the parent, followed by two separation-reunion episodes. During one of the separation episodes the parent was ‘virtually available’ to the child via a video link. Our results revealed three important differences. First, children left alone played longer in a strange room when their parent was virtually available to them compared to when the children were left alone with neither physical nor video contact with their parent. Second, younger participants sought physical contact with their parent less at the end of the video separation episode compared to when they were left entirely alone. Finally, the comparison between free play with video and free play with parent, revealed that the children exhibit a similar level of interactivity with their parent by video as they did in person.

**Conclusions/Significance:**

For young children a video connection can have many of the same effects as a physical presence. This is a significant finding as it is the first such empirical demonstration and indicates considerable promise in video communication as a tool to maintain family relationships when physical presence is not possible.

## Introduction

Since Bowlby [1] first introduced attachment theory, it has been accepted that physical proximity is necessary for young children to form and maintain a secure attachment with an adult. The notion that physical proximity is necessary for attachment seemed obvious (if not tautological) since a sufficient degree of interactivity seemed necessary for a child to form a close relationship with another person, and there was no modality for inter-activity other than physical proximity. The apparent case that physical presence is needed is strengthened by previous research indicating that young children have difficulties with traditional telephone conversations [2]. Now, with video communication, it is feasible for people to have real-time enriched communication without physical proximity. This interaction opportunity raises important and interesting questions about the extent, if at all, to which virtual proximity is enough for young children to maintain or possibly create relationships and establish a feeling of security with others.

Communication via the internet is a particularly popular means of maintaining contact with family members. In a recent survey of online users, almost half of over 6000 respondents indicated that the internet has improved relationships with their family overall, with 42% reporting that they had engaged in video communication with family or friends [3]. With more than 443 million active members of just one internet communication service [4], the popularity of this phenomenon for adults in society is undeniable.

Video communication is also increasingly prevalent in connecting young children and their relatives. Encouraging and supporting such interactions has been the development of purpose-built internet software and the marketing of specially designed devices [5]. A number of popular media reports attest to the burgeoning popularity of video communication for this purpose [6]–[8].

Advances in technology are often credited with dramatic social changes with potentially widespread (and often unpredictable and/or negative) effects on children's psychological and physical development [e.g. 9], [10]. Presumably, this is partially due to a shift away from patterns of behavior established over periods measurable in evolutionary time. Arguably however, video communication permits a greater amount of intergenerational contact more akin to what our forebears experienced than what is typical in modern society. Indeed, Western societies experienced pronounced changes in family and living arrangements during the 20^th^ century [11], and in today's society family members are often geographically separated [12], [13]. Many grandparents, for example, do not reside in close proximity to their grandchildren, and have limited face-to-face contact due to time and monetary restraints [14]. Research indicates that grandparent-grandchild relationships are beneficial and important for both generations [For a review see 15] and for those that are geographically separated, video communication may make such relationships possible.

Of potentially greater relevance to the focus of this paper, children are also separated from their parents for a variety of reasons. Almost half of divorces involve children, and more than half of young children (<5 years) from separated families see their non-custodial parent less than once a fortnight [12]. Extensive business travel also separates many families [16] as do the ‘Fly-in Fly-out’ work practices that are increasingly being implemented within the mining industry [17]. Longer-term separations can also arise when a parent is on a military tour of duty. With hundreds of thousands of Military Personnel from the US alone on Active Duty in foreign Countries [18] an increasing number of children await their parent's return, just to see them leave once again [19]. Furthermore, through the incarceration of a parent, many children are also separated from their mother [20] and/or father [21], and often for considerable periods of time. With many custodians unable or unwilling to take young children into the prison environment, a considerable number of these children have little or no contact with their incarcerated parent [22].

During such times of separation, video communication may provide these young children with the connection to their parent(s) and assist the children by psychologically lessening the distance caused by geographical separation.

Video communication is a seemingly rich experience, however a large body of research exists illustrating that young children treat people on video differently than people that they see face-to-face [E.G 23], and therefore it is important to establish the extent to which children that engage in video communication with someone that they have an emotional bond to, such as their parent, behave as if they are proximal to that person during the interaction. The answer to this question will provide insight into the potential of video communication as a means of establishing and maintaining relationships between young children and absent parents. Significant similarities in how children react emotionally to a virtual and physical presence would be suggestive of such potential. In contrast, if children respond to a virtual connection as if the children were physically alone, then maintaining relationships with children via video would be problematic at best.

Our experimentation strategy involved a modified version of the Separation and Reunion Paradigm [24]. Such paradigms have been used for decades to examine the behavior of children when they are separated from their parent [25], and can therefore be employed to investigate if a child feels separated from their parent when they are physically alone, but virtually connected via a video link. Establishing whether a virtual connection to their parent attenuates typical separation behaviors in a child will be an initial step in determining the extent to which the virtual connection can serve as a proxy for physical presence. We expected that for children as young as 17 months-of-age, the presence of their parent by video link would have effects similar to having a parent physically present. Therefore we hypothesized that children would remain content to be alone in the room for longer if their parent was virtually available to them and that children would use the virtual presence of their parent as a secure base for exploration [1], [24]. Children were also expected to behave differently during the reunion if they had virtual access to their parent during the separation and would be less inclined to seek comfort from or close proximity to their parent than when they did not have contact during the separation.

Conversely, an alternative hypothesis was that the presence of the parent via the video link would serve only as a reminder to the child that their parent was not actually present, and that children of some ages would find the virtual presence of their parent distressing rather than reassuring. This later hypothesis reflects previous research findings that demonstrate that toddlers treat face-to-face and online interaction differently [23].

## Materials and Methods

### Apparatus and Materials

The experiment was conducted in two adjoining lab spaces; a playroom and a computer room. A 175 cm×300 cm lab was set up as the play room and contained a couch and age appropriate toys including a drawing easel and pens, blocks, a train set and soft toys. The computer monitor sat on a shelf 1 m high positioned across the front wall with the computer box located out of reach of the children. An Ethernet cable connected the computer in the play room to a computer in the next room.

**Figure 1:**
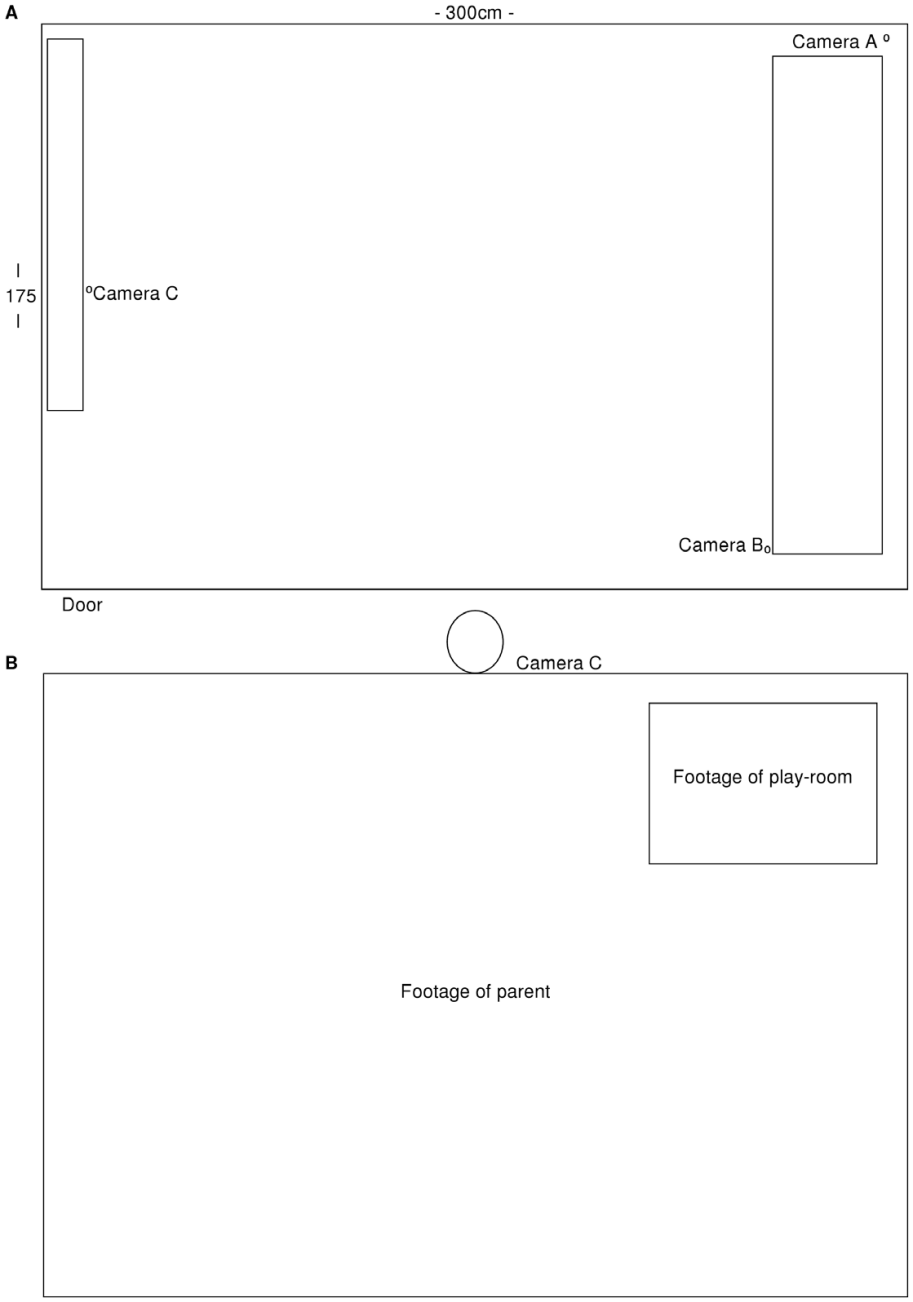
The physical arrangement of the playroom (A). Camera C was attached the computer monitor through which the video communication occurred (B).

Three cameras were positioned within the play room: Camera A was attached to the wall in the back left corner of the room, behind the couch; Camera B was attached to the couch arm in the back right corner of the room; and Camera C was attached to the computer monitor. See Figure 1(a). The video communication sessions were accomplished and recorded using the Apple Inc. software application iChat. The picture-in-picture feature was activated, resulting in the parent's webcam footage occupying the full screen of the playroom monitor with the playroom webcam footage presented in a small box in the top right corner of the monitor and the reverse on the parent's computer monitor. See Figure 1(b).

A sub-set of participants (n = 28) completed a questionnaire based on the Attachment Q Set (Waters, 1995). The questionnaire asked parents to rank on a 5 point Likert scale (−1, −.5, 0, +.5, +1) ranging from −1 = “not at all like my child”, to 1 = Very much like my child, the degree to which statements (borrowed from the Attachment Q Set cards), are generally characteristic of their child. For the current study only the specific behavioral characteristic that would be most descriptive of the prototypically secure child, have been considered and a *security score* was obtained by averaging those item scores. Comparable to the Q Set scoring, a score of +1 would reflect a perfect positive correlation to a prototypically secure child whereas a score of −1 would reflect a perfect negative correlation to a prototypically secure child. Positive scores would therefore indicate a child is securely attached. Results for the subset of participants whose parent completed this questionnaire revealed that all security scores corresponded to that of a secure child (*M* = .40, *SD* = .21). The findings of the current study are likely reflective only of securely attached children.

### Participants

Forty-five children participated in the experiment, however, as a result of technical problems in our apparatus four participants have been excluded from the analysis. Participants included in the analysis were 41 children aged 16.9–64.8 months (*M* = 35.2, *SD* = 14.3), including 21 females and 20 males. Ten children were aged 17 months <2 years, 7 were aged 2<2.5 years, 8 were aged 2.5<3 years, 8 were aged 3 years, 6 were aged 4 years and 3 were aged 5 years. The majority of children (85%) participated with their mother rather than their father. Participants were recruited through various avenues including advertisements online and in newsletters, and word-of-mouth referrals.

### Procedure

Each parent-child dyad participated in a separation and reunion protocol, which was based on that used previously by Ainsworth, Bell and Stayton [24]. The current protocol involved a free-play session followed by two separation-reunion episodes. During one of the separation episodes the parent was ‘virtually present’ to the child via a video link, allowing audio and visual real-time interaction. This will be termed the *Video separation episode*, and was counter-balanced to occur during either Separation 1 or Separation 2.

#### Free-play session

The parent and child were left alone in the play room for 10 minutes with no instructions other than to interact normally, and that the researcher would return after 10 minutes.

#### Video separation episode

The researcher entered the play room and asked the parent for their assistance in another room. The parent told their child that they would return soon and left the room. The researcher then took the parent into the next room from where they could communicate via the video link with their child. The parent was not provided with any further instructions and left to interact with their child via the video link for up to five minutes, or until the child showed signs of distress. The parent then returned to the playroom for the reunion episode.

#### Reunion episode 1

The parent returned to the play room without any further instructions. The reunion episode lasted five minutes before the researcher again entered the room and used the same instructions as before to facilitate the second separation episode.

#### Non-video separation episode

This episode was the same as the *Video separation episode* except that the child could not see or hear the parent. However the parent could see and hear the child.

#### Reunion episode 2

The parent returned to the play room without any further instructions. This episode lasted five minutes before the researcher then entered the room to conclude the session.

### Coding

#### Separation Episodes

From the video recordings of the episodes, the measure *contentment duration* was calculated to indicate the period of time that the child was content to be physically alone in the playroom, with and without virtual access to their parent via the video link. This was defined as the period of time (in seconds) that the child was physically alone in the room until they began to cry and continued crying for 10 seconds, or tried to leave the room. This variable had a maximum value of 300 seconds, as this was the maximum duration of each separation episode.

#### Reunion Episodes

To compare the reunion episodes following the video and non-video separation episodes, a *proximity* variable was created, noting whether or not the child moved towards the parent when they entered the room after each separation. This variable was only investigated in children under 3 years of age, as proximity seeking behavior is normal for such children and less usual for older children in Phase 4 of the development of attachment [1].

#### Comparison Across Episodes

To investigate the amount of time that the child played with toys and/or interacted with their parent, each 10 second period of the free-play, the video separation and the non-video separation episodes were coded. Only the first five minutes (I.e. 300 seconds) of the free-play session was coded to allow comparisons to be drawn between the free-play session and the separation episodes. Thus there were thirty 10-second intervals in each of the three periods (or less in the cases when the child remained in the room for less than the full 300 seconds of the separation episode).

Using the results of the 10-second interval coding the *play* criterion and the *interaction* criterion were defined as the number of 10-second periods during which the participant touched/played with the toys or otherwise interacted with their parent, respectively. The maximum score for any episode was 30. Variables were also computed to investigate the percentage of each episode that participants played, and percentage of each episode that participants interacted with their parent, since separation episodes were not all of equal duration. For example if a child played for 15 of the thirty 10-second intervals for which the child was in the room, the play percentage was 50%.

#### Inter-rater Reliability

Cohen's Kappa was computed to determine inter-rater reliability for *proximity (κ = .77, p<.001)*, and *play (κ = .72, p<.001)* with 40% of cases. Additionally there was an inter-coder correlation of 99% on *contentment duration (p<.001)*.

## Results

Preliminary statistical analyses indicated that the child's gender and previous video communication experience did not have any effects on the dependant variables. Therefore these variables have been eliminated from further analysis. Individual participant data can be found as Data S1.

Table 1 shows the median values of behavioral indicators for each episode, and results of statistical tests. All cases where participants were distresses immediately on separation, and thus the episode was terminated, are treated as having a duration of zero. Analyses where these cases were counted as missing did not alter the pattern of results.

**Table 1:** Medians of the Behavioral Indicators for Each Episode and Results of Wilcoxon Signed Rank Tests used to compare Behaviors across Episodes.

Measure	*P- value*	Condition	*Mdn*	*Z*
Duration of contentment	<.001	Video separation episode	300 sec	−3.81
		Non-video separation episode	79 sec	
Amount of play	<.001	Free-play episode	28 periods	−4.42
		Video separation episode	12 periods	
Amount of play	<.001	Free-play episode	28 periods	−5.26
		Non-video separation episode	4 periods	
Amount of play	<.001	Video chat separation episode	12 periods	−3.20
		Non-video separation episode	4 periods	
Percentage of play	<.001	Free-play episode	97%	−4.89
		Video separation episode	40%	
Percentage of play	<.001	Free-play episode	97%	−5.08
		Non-video separation episode	50%	
Percentage of play	.46	Video chat separation episode	40%	−0.74
		Non-video separation episode	50%	
Amount of interaction	.02	Free-play episode	27 periods	−2.4
		Video separation episode	24 periods	
Percentage of interaction	.162	Free-play episode	87%	−1.40
		Video separation episode	87%	
Proximity (Age <3yrs old)	.034	Non-video separation reunion	41.2%	−2.121
		Video separation reunion	5.9%	
Continued for the entire 300 s of the video separation episode	.033	Participants aged 2<2.5 yrs	42.9%	−2.135
		Participants not aged 2<2.5 yrs	82.4%	

The duration of contentment showed a marked difference across the two separation conditions with participants content to remain separated from their parent for significantly longer during the video separation episode compared to the non-video separation episode. In entirety, 85% of the participants were content for the whole video separation interval; whereas only 37% remained content when there was not a video link available.

We compared the amount of time that participants played during the free-play session, the video separation episode, and the non-video separation episode. A Friedman test indicated these conditions differed, χ^2^(2, *n* = 41) = 35.86, *p*<.001. Median values showed that participants played most during the free-play session, followed by the video separation episode and least during the non-video separation episode. Further planned comparisons using Wilcoxon Signed Ranks tests indicated that participants played significantly more during the free-play episode than during either the video or non-video separation episodes, and participants played significantly more during the video separation episode than during the non-video separation episode.

To control for the differences in the amount of time that participants remained in the room across the free-play and the two separation episodes, the percentage of time periods during which participants played while they were in the room was compared across conditions. A Friedman test showed that the percentage of time spent playing varied significantly, χ^2^(2, *n* = 41) = 35.58, *p*<.001. Median values showed that participants played for the greatest percentage of time during the free-play session, whereas they played for only about half the time in both separation episodes. Further planned comparisons indicated that participants played significantly more during the free-play episode than both the video and non-video separation episodes, however the separation episodes did not significantly differ.

Comparing the amount of time that participants spent interacting with their parent during the free-play and video separation episode showed that participants interacted to the same degree with their parent during the video separation episode as the free-play episode.

For the children under three years-of-age, we also tested for proximity seeking behavior after each separation episode. Significantly more children of these ages moved towards their parent during the reunion that followed the non-video separation than the reunion that followed the video separation.

To examine other possible age differences Kruskal-Wallis tests were performed to compare results across different age groups (Gp1, *n* = 10: 1.5<2 years, Gp2, *n* = 7: 2<2.5 years, Gp3, *n* = 7: 2.5<3 years, Gp4, *n* = 7: 3<4 years, Gp5, *n* = 9: 4<6 years). No significant age differences were found for three of the variables: the percentage of interaction during the video separation episode; the difference in contentment duration between the video and non-video separation episodes; and the duration of contentment for the non-video separation episode.

**Figure 2:**
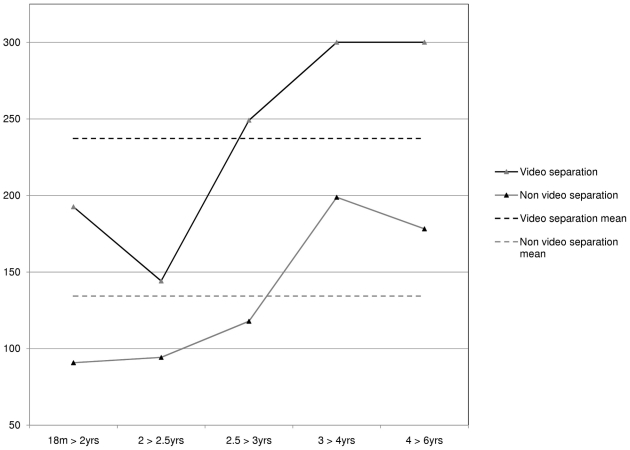
Age differences of the duration of contentment (in seconds) during the separation episodes.

Age groups differed significantly on *duration of contentment* for the video separation episode, χ^2^(4, *n* = 41) = 10.531, *p* = .032. The 3<4 year-old and the 4<5 year-old participants were content equally for the longest period of time (*Md* = 300 s) and the 2<2.5 year-old participants were content for the shortest period of time (*Md* = 70 s). See Figure 2.

Moreover, there was a significant difference across age groups in the number of participants who were content for the entire 300 seconds of the video separation, χ^2^(4, *n* = 41) = 10.676, *p* = .030. The 3<4 year-old and the 4<5 year-old participants were most likely to continue for the entire 300 seconds and the 2<2.5 year-old participants were the least likely.

## Discussion

The fundamental contribution of this research is the discovery that a parental presence via video link is sufficient to allow young children to feel secure in an unfamiliar environment. This empirical verification is crucial in considering the potential of video communication to play a role in the maintenance or formation of secure attachments.

Our conclusions are based on four measures of child behavior: duration of contentment, interaction with the parent, engagement in play, and response to reunion, all of which are common indices of attachment security [24].

Firstly, we consider duration of contentment, as that is arguably the most direct measure of how secure our participants felt when they were in the room. Children of all ages were content to be alone without a parent physically present significantly longer in the video separation than the non-video separation episode. Further, a greater percentage of children were content for the entire five-minute video separation than non-video separation. Interestingly almost all of the participants that did not remain content for the entire five minutes of the separation, showed signs of distress within two minutes of their parent leaving the room. Despite the brevity of the separations, it is probable that provided the opportunity, the content participants would have remained content far beyond the given five minutes, however future studies should address this question.

Secondly, our interaction measures revealed that children interacted slightly (though not significantly) more with their parent during the time that the parent was in the room than during the video separation episode. Notably however, there was no significant difference between the two episodes in the percentage of time that children interacted with parents. Thus, when children were content in the room they interacted with their parent as much in the video separation as they did when the parent was actually present. This is important because it suggests significant similarities between the quality of the virtual presence compared with real presence.

Thirdly, play was also an important measure in our study as it is an indicator of the extent to which a virtual parental presence can substitute for a physical secure base for exploration. Results indicated that children across all ages played for longer during the video separation than the non-video separation. However this result may arise, at least in part, because children could only play for the duration that they were in the room. Children who are content longer will have more time to play in the room and thus did so. Conversely, children who feel secure enough to use the video as a secure base for exploration will also be likely to stay in the room longer (and consequently play for longer).

In an attempt to partially control for duration of contentment we compared the percentage of time that they played for while they were content to be in the room. However, results revealed that children only played for about half of the time that they were in the room during both separations compared to when they were present with their parent. The failure to find differences in percentage of play between the video separation episode and the non-video separation episode may, however, be due to other differences in the nature of interaction in these two separation episodes.

Furthermore, the interaction measure should be considered in any interpretation of how much children played. Since video communication is still a relatively unusual activity in most households, the novelty of talking to a parent over a video link could be interpreted as “play” for many children. This might explain why the play percentage was not higher in the video separation condition than the non-video condition. That is, the play measure may be underestimating play during the video separation episode since we did not include video interaction with their parent within the measure play. Collapsing across interaction and play measures may be considered an option to overcome this discrepancy, however this variable would not allow comparison with the non video separation as there was no opportunity for interaction during that episode.

Additionally, there may be subtleties of how one interacts, plays and talks with a parent when they are actually present rather than virtually present that accounts for differences in play between these conditions. When playing and interacting within the presence of the parent, a child can interact, talk and play simultaneously and can readily assume that their parent is watching them. However, during the video separation, children may have paused more often in their play to turn and look at their parent on the monitor to interact with them. This may be especially so when children are not familiar with this medium, or when the video separation is occurring in a strange situation.

Finally, observations of the reunions demonstrated that the younger participants (under 3 years), were significantly more likely to move to contact their parent following the non-video separation than the video separation. This result is consistent with the literature that suggests that a child tends to seek proximity when attachment behavior is intensely activated [26], and that the non-video separation appears to have activated this behavior in the younger participants.

In sum, our results form compelling evidence that a parent's virtual presence is sufficient to increase the level of security felt by young children with pre-existing strong attachments in an unfamiliar environment. This is an important finding as it suggests that relationships between children and their parents could benefit from video communication when face-to-face contact is not possible.

It is conceivably possible that some children were happier during virtual communication because they were distracted from their separation by the novelty of talking to a parent via video. Although studies are underway to assess this possibility, it is important to note that a video of a person could also remind the child of the person's absence, rather than distract them from it. To some extent, the novelty issue was addressed through our analysis of the children's prior experiences with video communication. Children with less video communication experience would arguably be more likely to be subject to such a “novelty effect.” However, our results did not reveal effects of previous video communication experience on any dependent variable.

However, as video communication becomes more commonplace in society the role of prior experience with this medium may change, particularly if children are exposed to video communication at a very early age. These children may develop a level of expertise that allows them to better understand the precise extent to which a video can stand in for an actual person and where it suffers limitations. This might affect results in studies such as ours by attenuating the effect of age on duration contentment, or how play and interaction with the parent is negotiated via video. Investigation of which aspects of this communication medium are the most beneficial or problematic for young children is also required. As previous research has shown that young children have difficulties with traditional telephone conversations [2], it would be beneficial to extend the current protocol to investigate differences in a child's behavior with the availability of their parent via a video link compared to an audio stream and compared to actual presence. Future studies should also include children younger than those included in the present study, and also involve extended relatives rather than parents. Numerous anecdotal accounts report babies being introduced to absent grandparents and parents from an early age with regular interactions occurring via video link [8]. Many other such questions remain to be answered on how children negotiate and use this virtual medium when it has always been part of their life, and how it enables them to develop and maintain relationships with important others.

The study described here investigates the developmental effects of a relatively new technology, but somewhat ironically its usage has the potential to bring us closer to societal norms that existed in the past. Whereas only a few decades ago multiple generations often lived under one roof (or at least within the same neighborhood), extended families are increasingly separated by large distances so face-to-face contact is limited [11]. The evidence presented in this paper indicates that these video episodes may be sufficient for interaction that is meaningful to a young child. Continuing forward, researchers must ecologically determine if video communication provides a “real enough” experience to maintain relationships during longer-term separations, and ascertain the unrealized benefits to the children and parents, and potentially other members of the extended family. Our research paves the way for future studies that examine more directly the impact of video communication with children who may otherwise feel completely separated from relatives during times of physical absence.

## Supporting Information

Data S1 Supporting data.
(XLS)
